# Relationship between Socio-Emotional Competencies and the Overlap of Bullying and Cyberbullying Behaviors in Primary School Students

**DOI:** 10.3390/ejihpe11030049

**Published:** 2021-07-05

**Authors:** Juan Manuel Rodríguez-Álvarez, Santiago Yubero, Raúl Navarro, Elisa Larrañaga

**Affiliations:** 1 Education and Youth Department of the Regional Government of Madrid, Plaza de la Asamblea de Madrid, 28018 Madrid, Spain; 2 Department of Psychology, Faculty of Education and Humanities, University of Castilla-La Mancha, Avda de los Alfares, 42, 16071 Cuenca, Spain; santiago.yubero@uclm.es (S.Y.); raul.navarro@uclm.es (R.N.); 3 Department of Psychology, Faculty of Social Work, University of Castilla-La Mancha, Avda de los Alfares, 42, 16071 Cuenca, Spain; elisa.larranaga@uclm.es

**Keywords:** socio-emotional competencies, cyberbullying, bullying, primary education, gender

## Abstract

Digital life forms part of daily reality for young people. For this reason, traditional bullying in school has been reproduced in the online environment, resulting in an overlap of off- and online bullying. Research on socio-emotional competencies and bullying is revealing interesting results among students in secondary schools. However, studies involving primary school students are much scarcer. In addition, the majority of studies have been carried out based on an understanding of socio-emotional competencies as a unidimensional construct. In the present study, we examined the overlap between off- and online bullying victimization and the influence of the factors comprising socio-emotional competencies on this overlap. Participants comprised 1130 students (49.7% were boys and 50.3% were girls) from the fifth and sixth grades at 15 schools in the autonomous communities of Madrid and Castilla-La Mancha (Spain). The results indicate a high rate of overlap between off- and online bullying victimization, without significant gender differences. Poor relationship skills in boys and low self-management in girls were associated with being a victim of both traditional bullying and cyberbullying. The conclusions point to an interesting line of intervention and prevention, establishing a framework of confluence for social and emotional variables within the primary education context.

## 1. Introduction

Bullying has been defined as an aggressive behavior that is repeated over time within a relationship in which there is an inequality of power and in which the victim is isolated. When this aggressive behavior is inflicted through electronic devices, it is called cyberbullying [1]. Bullying is a problem that schools confront by necessity, given its serious consequences for academic performance [2] and health [3].

Difficulty in establishing relationships has been considered a risk factor for bullying [4,5]. In adolescents, socio-emotional competencies have been shown to predict positive interpersonal relationships, including peer relationships [6,7]; it has also been observed that they improve the atmosphere in school [8] and prevent risk behaviors such as technological abuse [9] or violence [10]. It has been shown that students high in socio-emotional skills are good communicators, know how to negotiate conflicts constructively, seek help when they consider it necessary, and adopt responsible social behaviors [6,7].

The young people of today were born into the era of the Internet, and digital life is part of their daily reality; fundamentally, social networks have generated new patterns of interaction and communication [11]. Previous studies have indicated that the Internet is, at a basic level, a prominent interaction space for personal relationships [12]. Social networks allow interaction in a cheap, simple, and entertaining way, which leads young people to adopt their use from the earliest age at which they have access to the Internet. In Spain, the National Institute of Statistics reported in 2020 that almost 86.7% of children from 10 years old were Internet users [13].

On the other hand, numerous studies confirm that relationships established in the offline world are continued into the online world [14]. This can lead to the transfer of bullying behaviors from the school space to the online space, giving rise to an inappropriate use of social networks [15].

Victimization at an early age can foster maladaptive development patterns, which become a risk factor for victimization in later stages of development [16]. According to the results of longitudinal studies, the victimization suffered in the final years of primary education continues into secondary school [17] and has serious consequences for students [5,18]. Previous studies have reported that more than half of adolescent victims of bullying experienced victimization in primary education [19,20]. However, studies investigating the preadolescent stage remain scarce.

### 1.1. Cyberbullying in Primary Education

The majority of research on cyberbullying has been carried out with adolescent subjects [21]. It has, as such, been considered that cyberbullying is more common in adolescence due to superior access to new technologies; yet, it is known that young children are also making use of Internet-based applications with great frequency. However, very few studies have focused exclusively on students in primary education. In Machimbarrena and Garaigordobil’s review of research in the last five years, only 20 studies used samples of students under 14 years of age [22].

Although cyberbullying is more frequent in adolescence [23], various studies have reported cyberbullying behavior in the final years of primary education; in fifth grade and sixth grade, or from 10 to 12 years of age [24,25,26]. Regarding gender, results are inconclusive; while some studies indicate higher victimization in girls [24], others report no gender differences [27]. Among the recent studies examining cyberbullying prevalence among primary school students, Aizenkot [28] reported that 7.8% of the Israeli participants were cyber-victimized in their Whatsapp group chat. Escortell et al. [29], assessing 15 electronic harassment behaviours in a sample of 548 Spanish students aged 10 to 13 from Alicante, found that 18% of the participants reported suffering cybervictimization. In a series of studies with students in years 3 to 6 of primary school in Catalonia (Spain), Sidera et al. found that 14.4% of the participants have suffered at least one online aggression in the last month [30] and 4.4% were cyber victims with a minimum frequency of once or twice a month [31].

### 1.2. Overlap of Off- and Online Bullying

Research has indicated that it is possible for victims to be attacked simultaneously with different forms of bullying [32]. The results of many studies suggest that a coexistence of traditional bullying (offline) with cyberbullying (online) is also found in primary education [33,34,35]. Although research has reported a greater frequency of traditional bullying than cyberbullying [3,36,37], a strong relationship has been found between victimization in offline and online contexts. Study results suggest that online victimization tends to occur together with victimization in a physical environment [38]. In fact, only a small percentage of students are victims of cyberbullying exclusively [39,40].

For these reasons, we consider the overlap of off- and online bullying in young people to be an important area of research with regard to providing appropriate tools for early prevention.

### 1.3. Socio-Emotional Competencies and Cybervictimization

Socio-emotional competencies refer equally to emotional skills, understanding and attitudes, on the one hand, and to social competencies, on the other [41]. The former includes responsible decision-making, relationship skills, self-management, social awareness, and self-awareness [42]. The Collaborative for Academic Social and Emotional Learning provided the conceptualization of the factors linked to socio-emotional competencies [43]. Responsible decision-making refers to the capacity to resolve social problems, taking into consideration the needs of others; it includes making respectful and morally acceptable decisions regarding one’s behavior and concerning one’s relationships with others. Relationship skills refer to the capacity to establish and maintain healthy friendships, listen to others, work cooperatively, manage conflict constructively, and help others. Self-management relates to one’s abilities in regulating emotions and behavior. Social awareness has to do with the capacity to understand the behavior and perspectives of others; showing empathy. Self-awareness refers to the capacity to evaluate personal strengths and weaknesses, but also the ability to identify our emotions and thoughts, and how these can affect our behavior.

Research into the relationship between bullying and socio-emotional competencies is showing highly interesting results in secondary school students. The majority of such studies have been carried out based on an understanding of socio-emotional competencies as a unidimensional construct. Embracing a multidimensional understanding of socio-emotional competencies, Yang et al. note that social awareness is positively associated with bullying victimization, while self-management and relationship skills showed a negative association with said victimization, and responsible decision-making had no significant associations [44]. With respect to cyberbullying, research results are inconsistent [45]. Some studies have reported lower socio-emotional competency in the victims of cyberbullying [46], while other studies have found no significant differences—in respect of socio-emotional competencies—between students who are uninvolved in cyberbullying and those who are victims [47,48]. To date, there have been no studies that analyze the relationship between the off- and online bullying overlap and factors associated with socio-emotional competencies.

Many studies have demonstrated gender differences in socio-emotional competencies, with women presenting higher levels [49]. We consider it important to include the gender variable in the analysis of the relationship between socio-emotional competencies and cyberbullying.

Previous research shows that further progress is required in the study of the relationship between socio-emotional competencies and cyberbullying [45,48]. Building upon the work of the previous review, our objectives are: first, to study the overlap of off- and online bullying, and, second, to analyze the influence of socio-emotional competencies on victimization within the overlap of these two types of bullying (offline and online).

For this purpose, we propose four hypotheses: (H1) There will be an overlap of off- and online bullying and its prevalence will be greater than the prevalence of online bullying alone; (H2) this will occur in a similar way in boys and in girls. Concerning the second objective, (H3) we expect that relationship skills, self-management, and social awareness will be associated with the off- and online bullying overlap, and (H4) gender differences are expected in this regard.

## 2. Material and Methods

### 2.1. Participants

Participants were randomly selected from 13 schools in the autonomous communities of Madrid (46.4%) and Castilla-La Mancha (53.6%). The study sample included 1130 students from the fifth and sixth grades of primary school, aged between 10 and 12 years (*M* = 10.78, *SD* = 0.70), of which 49.7% were boys and 50.3% girls. Fifth grade students accounted for 51.6% of the sample, and sixth grade for 48.4%.

### 2.2. Instruments

To measure bullying and cyberbullying, we used the Bullying, Harassment, and Aggression Receipt Measure (BullyHARM) [50]. It is composed of 14 Likert-type items with four response options from 0 to 3 (0 = it has not happened to me, 1 = it has happened to me 1–2 times, 2 = it has happened to me at least once in the last week, and 3 = it has happened to me 2 or more times in the last week). Students were asked to rate the frequency of their involvement in certain behaviors during the previous month. The measure provides information on physical bullying (five items, e.g., “Pushed or pulled on me”), verbal bullying (three items, e.g., “Called me a bad name”), social bullying (three items, e.g., “Tried to turn people against me”), and cyberbullying (three items, e.g., “Posted something bad about me on the Internet”). In the present study, the reliability was adequate: physical bullying (Ω = 0.84), verbal bullying (Ω = 0.87), exclusion (Ω = 0.86), and cyberbullying (Ω = 0.81).

Socio-emotional competencies were collected using the Delaware Social and Emotional Competencies Scale–Student (DSECS-S) [51], which had already demonstrated its suitability for use with primary school students [44]. The DSECS-S consists of 12 items; three items in each of the four factors: responsible decision-making (e.g., “I blame others when I’m in trouble”), relationship skills (e.g., “I am good at solving conflicts with others”), self-management (e.g., “I can control how I behave”), and social awareness (e.g., “I think about how others feel”). Students had to respond to each item using a Likert-type scale from 1 = Never to 4 = Always. In addition, the McDonald coefficient was employed, obtaining adequate reliability values: responsible decision-making (Ω = 0.71), relationship skills (Ω = 0.82), self-management (Ω = 0.85), and social awareness (Ω = 0.81).

### 2.3. Procedure

Taking account of ethical considerations, we proceeded first to obtain the informed consent of the school students’ parents. Of these families, 0.9% did not respond, and therefore their children did not participate in the study.

The questionnaire was administered by members of the research team, and the students answered it within classrooms, with the prior agreement of the school leaderships and the teachers. The objective of the study was explained to the students, and they were informed about the voluntary nature of their participation and the anonymity of their responses. The approximate mean response time was 20 min. All the procedures performed in this study were in accordance with the Declaration of Helsinki.

### 2.4. Analysis of the Data

First, we analyzed the overlap between victimization in cyberbullying behaviors (online bullying) and victimization in physical, verbal, and social bullying (offline bullying), using a contingency analysis.

To establish the study’s overlap group, students who had been victimized both offline and online were considered. Using the chi-square test, we studied both the prevalence of the off- and online bullying overlap and the comparison by gender. Subsequently, we performed a Pearson correlation analysis in order to determine the relationships between socio-emotional competencies and the bullying overlap, and we carried out a Student’s *t* test to verify the existence of differences in socio-emotional competencies according to gender. Finally, we performed a multinomial logistic regression analysis to analyze the predictive value of socio-emotional competencies, independently, for gender. All analyses were performed using the SPSS statistical package (version 24) at a significance level of 0.05.

## 3. Results

### 3.1. Prevalence of the Off- and Online Bullying Overlap

Table 1 shows the overlap of the victimization represented by each cyberbullying item with victimization by physical, verbal, and social bullying. The results show statistical significance in boys for every online bullying item in relation to the three forms of offline bullying that were analyzed. In girls, the item for posting something on the Internet did not reach significance.

Among the sample, 68% of students reported having been a victim of offline bullying, and 6.6% in the case of online bullying. When the two types of bullying were analyzed together, 61.4% of students were found to be offline bullying victims, 0.2% online bullying victims, and 6.4% victims of overlapping off- and online bullying. With regard to gender, there were no significant differences (χ^2^ = 5.85, *p* = 0.119). In boys, 63.3% were victims of offline bullying, 0.4% were victims of online bullying, and 7.1% were victims of overlapping off- and online bullying. Among girls, 59.4% were offline bullying victims, none reported being exclusively bullied online, and 5.9% were victims of overlapping off- and online bullying.

### 3.2. Relationship between Socio-Emotional Competencies and the Bullying Overlap

The Pearson correlation analysis showed that there were statistically significant correlations in a positive direction between all the components of socio-emotional competencies and, in a negative direction, between these components and the off- and online bullying overlap (Table 2). As may also be observed in Table 2, there were statistically significant differences by gender in four components of socio-emotional competencies, which were found to be higher in girls.

### 3.3. Relationship with Factors of Socio-Emotional Competencies

Regression analysis was performed with the aim of explaining victimization in overlapping off- and online bullying through socio-emotional competencies. Analyses by gender were carried out independently. The results obtained (Table 3 and Table 4) confirm the relationship with socio-emotional competencies. Specifically, relationship skills (OR = 0.41) were found to explain the off- and online bullying overlap in boys, and self-management (OR = 0.31) was found to explain said overlap in girls.

## 4. Discussion

The first objective of our research was to study the off- and online bullying overlap in primary school students, while the second objective was to analyze the link between the off- and online bullying overlap and the factors pertaining to socio-emotional competencies. The majority of studies have been carried out with secondary school students and have been based on a unidimensional understanding of socio-emotional competencies. We consider it important to know which specific factors of socio-emotional competencies can be determining with respect to the simultaneous occurrence of offline and online bullying victimization.

With regard to the first objective, our results converge with those of previous research, showing a greater frequency of victimization in traditional bullying (offline bullying) than in cyberbullying (online bullying) [3,36,37]. While 6.6% reported being victim to online bullying, around ten times more students—68%—reported experiencing offline victimization. When off- and online victimization were analyzed together, the results offer confirmation of the bullying overlap, with the prevalence of overlapping offline and online bullying being higher than the prevalence of exclusively online bullying (H1). Consistent with the results of previous studies, only 0.2% of students reported having been a victim of exclusively online bullying, compared to the 6.4% who reported having been a victim of off- and online bullying [39,40].

These results confirm the bullying overlap [33,39] and demonstrate a high rate of overlap between online bullying and offline bullying. Previous research has found that adolescents who were victimized through various forms of traditional bullying exhibited more risky behavior linked with the use of technology [52]. In primary school students, the same trend is observable in relation to cyberbullying, confirming the overlap of victimization in physical and online settings in the final years of primary education [35]. These results indicate that offline interpersonal relationships are continued into the online environment [19]. The confirmation of the off- and online bullying overlap supports the understanding of bullying as a single phenomenon that can occur in various forms, including cyberbullying [3]. These considerations highlight the need to jointly analyze all forms of bullying in order to properly understand the complexity of this phenomenon. 

No significant differences were found with respect to gender (H2), although it is important to note that none of the girls participating in the study reported being victimized exclusively online. Among boys, 7% suffered both off- and online victimization, compared to 6% of girls. Previous research has indicated the relevance of gender identity to differences in bullying behavior [53,54]. It would be interesting to analyze its influence on cyberbullying behaviors and on the off- and online bullying overlap.

Regarding the second objective, the correlational analysis confirms the relationship between factors of socio-emotional competencies and victimization in overlapping off- and online bullying. The results indicate higher levels in girls for all the factors of socio-emotional competencies. This difference has previously been indicated in other studies [49].

In the regression analysis, only relationship skills in boys and self-management in girls were found to be significant. This confirms gender differences (H4) but only partially supports H3. In the study by Yang et al., social awareness is positively associated with bullying victimization, whereas in our study it did not reach significance [44]. This could be due to the age of the study subjects; our study involved the participation of children between 10 and 12 years. Meanwhile, self-management and relationship skills were found, in our study, to have a significant negative association with the off- and online bullying overlap, as in the research by Yang et al. [44]. Here, our study provides the gender analysis, entering one competency into the model for boys (relationship skills) and another into the model for girls (self-management).

As we have already noted, relationship skills refer to the capacity to establish and maintain healthy friendships and to resolve conflicts [43]. Students with greater relationship skills are more accepted by their classmates and have more friendships [55]. Consistent with the above, studies have noted bullying victims to be lacking in social skills and social adjustment [46,56]. In addition, previous research has indicated that their low capacity to defend themselves and to resolve conflicts can turn victims into easy targets for aggressors, who are able to take their aggression from physical settings into online settings [57]. The results of this study indicate that boys with poor social skills are more likely to be victims of overlapping off- and online bullying.

Among girls, self-management was found to be significant. Self-management refers to the ability to regulate one’s emotions [43]. High competency in self-management is associated with fewer behavioral problems and with close social relationships [58]. Previous research has indicated that low emotional efficiency increases the likelihood of being a victim of bullying [59], and has also included emotional difficulties within the description of victims [60].

The results of numerous studies have suggested that socio-emotional competencies can be protective factors against bullying in adolescence [15,48]. The results of this study confirm that socio-emotional competencies can also be a protective factor against victimization in preadolescence. In addition, our research directs us to focus on intervention in two specific factors: relationships skills and self-management, with consideration to the gender of the students.

If relationship skills and self-management are related to victimization in overlapping off- and online bullying among primary school students, then promoting these competencies could protect children from becoming victims. It is necessary to influence the intervention among this age group, including through programs adapted to their period of development [22]. Furthermore, it must be considered that prevention and intervention work with children is more efficient when it is carried out before problem behaviors become part of children’s normal repertoire and can be considered normalized behaviors [61]. In addition, victimization at an early age is a risk factor for victimization during subsequent stages of education [16,17,19,20]. Hence, intervention in the primary education stage can have preventative effects concerning bullying in secondary education.

Practical implications should also consider participants’ gender. Regarding boys, prevention programs should develop abilities to establish/maintain positive relationships and solve conflicts. In this sense, positive and healthy conflict resolution strategies should be reinforced to avoid aggressive strategies, such as win-win strategies. Regarding girls, prevention programs should address emotional regulation, working to recognize and control emotions to enhance well-being and prevent bullying. Emotion regulation programs may help to recognize unpleasant emotions and help students to deal with them in a positive way. For example, resilience has been found to be related to better outcomes when facing poly-bullying victimization [62] and it could be promoted from an early age.

This study presents limitations that should be taken into account in future research. Firstly, the use of a cross-sectional design limits conclusions regarding causality, meaning that it will be necessary to confirm the relationships that we found using longitudinal studies. We should also be cautious concerning the problems of social desirability and subjectivity that are entailed by the application of questionnaires. Previous research has indicated that the number of items that comprise the measure used has an influence on the results obtained [48], meaning that it is difficult to compare results with other investigations. Nonetheless, the values that we obtained from our analyses are similar to those resulting from previous studies [15,48]. Regarding the area of investigation, our study focuses exclusively on victimization. Other studies have demonstrated significant relationships between emotions and aggressive behavior [63], as well as between socio-emotional competencies and aggressive behavior [46,47,48]. Future research should be directed toward the analysis of the relationship between socio-emotional factors and the bullying overlap in all intervention contexts. It may also be of interest to analyze the connection with resilience, which has been shown to be relevant as a mediating variable of victimization [64].

In spite of its limitations, the present study contributes to research on cyber-coexistence. It is the first study to analyze the link between socio-emotional competencies—understood as a multidimensional construct—and the off- and online bullying overlap. We believe that this study provides suggestive data for early intervention against bullying, highlighting the need to strengthen specific factors of socio-emotional competencies that predict victimization in overlapping off- and online bullying. Our results point to an interesting line of intervention, establishing a confluence framework for social and emotional variables.

There is existing evidence of the importance of working on socio-emotional competencies in the curriculum during the primary education stage [7,65]. Teachers are key actors in carrying out interventions that turn schools into safer places for all pupils; however, teachers and schools, by themselves, are not able to implement the support that these children need if other social agents do not collaborate. The entire educational community has to jointly confront the problem of bullying. In this respect, professionals in socio-educational intervention need to understand the school institution as a strategic space for the integration of younger generations, providing resources and educational programs from different educational contexts that can complement the work of schools [66].

## 5. Conclusions

Poor relationship skills in boys and low self-management in girls account for a part of the explanatory percentage for being a victim of bullying in both physical and online settings. It is important to highlight that, according to the results of longitudinal studies, victimization suffered in the final years of primary education continues into secondary school [17] and has serious consequences for students [18]. In this context, we consider the provision of socio-emotional tools to be important to its early prevention.

## Figures and Tables

**Table 1:** Contingency of cybervictimization behaviors (online) over traditional bullying victimization (offline).

Variables	Total			Boys			Girls		
	Yes	No	χ^2^ (1)	Yes	No	χ^2^ (1)	Yes	No	χ^2^ (1)
They posted something bad about me on the Internet									
Physical bullying	2.5	0.7	5.36 **	3.9	0.4	6.83 **	0.8	1.0	0.08
Verbal bullying	2.7	0.7	7.13 **	4.0	1.1	4.81 *	1.5	0.3	2.13
Social bullying	2.9	0.8	7.94 **	4.5	1.2	5.98 *	1.6	0.3	2.63
They sent me a hurtful email or message									
Physical bullying	4.7	0.7	16.04 ***	5.2	0.4	9.69 ***	4.2	1.0	6.02 **
Verbal bullying	4.9	0.9	16.89 ***	5.0	1.4	5.89 *	4.8	0.3	11.83 ***
Social bullying	5.7	0.8	24.23 ***	6.3	1.2	11.18 ***	5.2	0.3	13.80 ***
They made hurtful comments about me on the Internet									
Physical bullying	4.0	0.6	14.57 ***	5.5	0	12.96 ***	2.3	1.0	1.53
Verbal bullying	4.0	0.9	11.98 ***	5.0	1.4	5.89 *	3.0	0.3	6.27 *
Social bullying	4.4	0.9	14.50 ***	5.8	1.5	8.15 **	3.2	0.3	7.42 **

Note. * *p* < 0.05, ** *p* < 0.01, *** *p* < 0.001.

**Table 2:** Pearson correlations, means, and standard deviations according to gender; Student’s *t*.

Variables	1	2	3	4	5
1. Decision-making	--	0.68 ***	0.59 ***	0.64 ***	−0.15 ***
2. Relationship skills	0.65 ***	--	0.60 ***	0.62 ***	−0.19 ***
3. Self-management	0.55 ***	0.49 ***	--	0.87 ***	−0.14 ***
4. Social awareness	0.57 ***	0.52 ***	0.86 ***	--	−0.18 ***
5. Bullying overlap	−0.10 **	−0.14 ***	−0.15 ***	−0.12 **	--
*M* boys	3.39	3.40	3.21	3.20	
*SD* boys	0.57	0.64	0.69	0.68	
*M* girls	3.51	3.49	3.34	3.39	
*SD* girls	0.49	0.55	0.60	0.56	
*t*	−3.74 ***	−2.43 *	−3.40 ***	−4.89 ***	

Note. Boys above the diagonal, girls below the diagonal. Measurement scale: from 1 to 4. * *p* < 0.05, ** *p* < 0.01, *** *p* < 0.001.

**Table 3:** Model for boys.

Variables	Offline Bullying		Online Bullying		Overlap	
	OR	IC 95%	OR	IC 95%	OR	IC 95%
Decision-making	0.71	0.41–1.22	1.93	0.02–16.82	0.85	0.34–2.11
Relationship skills	0.73	0.45–1.21	12.42	0.04–38.61	0.41 *	0.18–0.91
Self-management	0.86	0.47–1.58	2.10	0.03–13.37	1.09	0.37–3.19
Social Awareness	0.94	0.49–1.79	0.08	0.00–6.22	0.57	0.18–1.83
-2 LL	602.10					
Nagelkerke R^2^	0.08					

Note. The reference category is No intervention in bullying. Model χ^2^ = 33.86, gl= 12, *p* < 0.001. * *p* < 0.05.

**Table 4:** Model for girls.

Variables	Offline Bullying		Overlap	
	OR	IC 95%	OR	IC 95%
Decision-making	0.66	0.38–1.15	1.05	0.36–3.06
Relationship skills	0.76	0.46–1.24	0.46	0.20–1.08
Self-management	0.77	0.40–1.47	0.31*	0.10–0.95
Social awareness	1.15	0.57–2.33	1.58	0.45–5.58
-2 LL	540.12			
Nagelkerke R^2^	0.06			

Note. The reference category is No intervention in bullying. Model χ^2^ = 24.86, gl = 8, *p* < 0.001. * *p* < 0.05.

## Data Availability

The data that support the findings of this study are available on request from the corresponding author. The data are not publicly available due to data containing information that could compromise the privacy of research participants.
